# Editorial: Big data for biomedical research of inflammatory diseases

**DOI:** 10.3389/fphar.2023.1287616

**Published:** 2023-09-20

**Authors:** Jingxin Mao, Fancheng Meng, Guoze Wang

**Affiliations:** ^1^ Chongqing Medical and Pharmaceutical College, Chongqing, China; ^2^ College of Pharmaceutical Sciences, Southwest University, Chongqing, China; ^3^ The Key Laboratory of Environmental Pollution Monitoring and Disease Control, School of Public Health, Ministry of Education, Guizhou Medical University, Guiyang, China; ^4^ Department of Endocrine and Breast Surgery, The First Affiliated Hospital of Chongqing Medical University, Chongqing, China

**Keywords:** big data, biomedical research, healthcare, inflammation disease, multi-omic

Inflammatory diseases, which include infectious and autoimmune diseases, malignant tumors, cardiovascular diseases, diabetes, and other chronic noninfectious diseases (NCDs) that fall under the category of inflammatory diseases, have emerged as a major threat to the health of the global population due to increasing urbanization, the aging process, and lifestyle changes (Lontchi-Yimagou et al., 2013; Brusini et al., 2020). These illnesses are becoming serious public health issues that pose a serious threat to human health and socioeconomic sustainability (Pearson et al., 2003; Calder et al., 2009). New methods for disease prevention, diagnosis, and therapy will be developed by researching the mechanisms of inflammation occurrence and progression among these diverse diseases.

Biomedical research of pharmacology and medicine in the 21_st_ century is undergoing a change from conventional laboratory methods to new digital methods facilitated by the application of high-performance computing and big data analytic methods (Costa, 2014; Dash et al., 2019). The challenges of big data in biomedical research of pharmacology and medicine include big data capturing, analysis, information retrieval, transfer and visualisation, and information privacy protection (Elgendy and Elragal, 2014). The research domains have encompassed the amount, variety, and velocity of big data in addition to other pertinent technology methods such high throughput data analysis, machine learning, natural language processing, medical knowledge representation, and healthcare decision making (Bui et al., 2017). When these techniques are used simultaneously on the same biospecimen, they produce information with astounding molecular accuracy that can be enhanced by various imaging data types, biosignals, or clinical records (Loh et al., 2022).

Consequently, the title of this Research Topic “*Big data for biomedical research in inflammatory diseases*” was aimed to better provide the academic frontiers in computational methods for biomedical research of pharmacology and medicine in healthcare big data. It intends to bring together new findings of inflammation disease research related to big data, bioinformatics, and precision medicine. Papers having strong linkages to inflammatory disease-associated multi-omic integration, big data analytics, and health monitoring/diagnostic applications are encouraged. In addition, experimental verification that related to inflammation disease except big data analysis for biomedical research is considered necessary. Thus, we totally collected six original research articles by well-recognized authors on related fields.

Hui et al. explored the underlying mechanism of action of Emodin (EMO) against rheumatoid arthritis (RA) using network pharmacology approach. The result revealed that EMO inhibits inflammatory response of rheumatoid arthritis (RA) by targeting high-mobility group box 1 protein (HMGB1), signal transducer and activator of transcription 1 (STAT1), early growth response protein 1 (EGR1), nuclear receptor subfamily 3 group C member 1 (NR3C1), epidermal growth factor receptor (EGFR), mitogen-activated protein kinase 14 (MAPK14), caspase 3 (CASP3), caspase 1 (CASP1), interleukin 4 (IL4), interleukin 13 (IL13), inhibitor of nuclear factor kappa-B kinase subunit beta (IKBKB), fibronectin 1 (FN1), and monocytes/macrophages.

Yin et al. identified that Hect, uba, and wwe domain containing 1 (HUWE1), which plays a critical role in CD4^+^ T-cell activation and Sjögren’s syndrome (SS) pathophysiology. The findings demonstrated that HUWE1 may regulate CD4^+^ T-cell activation and SS development by modulating ATP-binding cassette transporter A1 (ABCA1)-mediated cholesterol efflux and presents a promising target for SS treatment.

Fan et al. demonstrated that macrophage module genes by weighted gene co-expression network analysis (WGCNA) in RNA sequencing (RNA-seq) data from chronic obstructive pulmonary disease (COPD), and then identified macrophage marker genes by comprehensive analysis of single-cell RNA sequencing (scRNA-seq) data from COPD macrophages. It revealed the vital role of macrophage ferroptosis in COPD, and novel biomarkers which including suppressor of cytokine signaling 1 (SOCS1) and small heat shock protein beta-1 (HSPB1) may be involved in the pathogenesis of COPD by regulating macrophage ferroptosis.

Tu et al. explored hub genes related to the abundance of immune cell infiltration in sepsis. The GEOquery package was used to download and organize data from the Gene Expression Omnibus (GEO) database. Animal experiments were carried out *in vivo* to detect the concentration and the expression of several immune factors. It was found that some immune cells showed significant differences between sepsis samples and control samples.

Zeng et al. revealed a mechanism that indicates celastrol can alleviate hepatocellular carcinoma (HCC) indirectly by regulating gut microbiota-mediated bile acid metabolism and downstream signaling. In their study, they established a rat model of orthotopic HCC and conducted 16S rDNA sequencing and UPLC-MS analysis. The results showed that celastrol may regulate gut microbiota and liver bile acid metabolism, inhibit the interaction between farnesoid X receptor (FXR) and retinoid X receptor alpha (RXRα) in the liver, induce mechanistic target of rapamycin (mTOR)/ribosomal protein S6 kinase B1 (S6K1)-related cell cycle G0/G1 phase arrest, and finally alleviate the proliferation of HCC.

Li et al. investigated the role of M1 macrophages in diabetic foot ulcers and related immune regulatory mechanisms. Diabetes foot ulcers (DFUs) are characterized by immune infiltration of M1 macrophages observed in foot skin, in which immune-associated genes (IRGs) play a prominent role. Based on the bio-informatics analysis of single-cell RNA (scRNA) and high-throughput sequencing data, it was revealed that M1 macrophages may be a influencing factor in the non-union of DFUs. Additionally, nuclear factor (erythroid-derived 2)-like 2 (NFE2L2), may be involved in the regulation of IRG expression within M1 macrophages.

Taken together, these above studies provide some of the interesting results and add new knowledge on the big data for biomedical research in inflammatory diseases. We are grateful to “Frontiers in Pharmacology” for giving us the opportunity to propose this Research Topic and invite a lot of authors in the related field. We also want to thank the editor for their quality control of the article, the reviewers for their valuable time and insightful suggestions, and the journal for providing this important platform.
